# Effectiveness of AOS–iron on iron deficiency anemia in rats

**DOI:** 10.1039/c8ra08451c

**Published:** 2019-02-11

**Authors:** Hong He, Qun Huang, Cancan Liu, Shirong Jia, Yiwei Wang, Fengping An, Hongbo Song

**Affiliations:** College of Food Science, Fujian Agriculture and Forestry University Fuzhou Fujian P. R. China sghgbode@163.com pingfengan@163.com +86-591-83789294 +86-591-83789294; Fujian Provincial Key Laboratory of Quality Science and Processing Technology in Special Starch Fuzhou Fujian P. R. China

## Abstract

Iron deficiency anemia (IDA) is one of the most serious nutritional problems. This study aimed to evaluate the therapeutic effects of a novel agar oligosaccharide–iron complex (AOS–iron) on rats with IDA, such as iron supplementation and recovery of antioxidant ability. Eighty-four weaned male SD rats were randomly divided into a normal control group (*n* = 12), which was fed with a standard diet, and an anemia model group (*n* = 72), which was fed with an iron-deficient diet for 4 weeks to establish a model of IDA. After the model was established, the rats with IDA were divided into six groups, namely, an anemia model group, a ferrous gluconate group, a ferrous sulfate (FeSO_4_) group, and low-dose (LD), medium-dose (MD) and high-dose (HD) AOS–iron groups, and fed with an iron-deficient diet and different iron supplements for 4 weeks, respectively. The results showed that HD AOS–iron exerted a significant restorative effect by returning blood parameters to normal levels in rats with IDA, including hemoglobin, red blood cells, hematocrit, mean cell volume, mean cell hematocrit, mean cell hemoglobin concentration, serum iron, total iron binding capacity, transferrin saturation, and serum ferritin. A histological analysis suggested that the liver morphology in the MD and HD AOS–iron groups was similar to that in the normal group. Furthermore, MD and HD AOS–iron improved antioxidant activities in the serum and liver. In general, high-dose (the same dose as those of ferrous gluconate and FeSO_4_) AOS–iron exhibited the best effects in terms of iron supplementation and antioxidant activities. The present findings showed that AOS–iron might be a potential new iron supplement.

## Abstract 2

Iron deficiency anemia (IDA) is one of the most serious nutritional problems.

## Graphical abstract

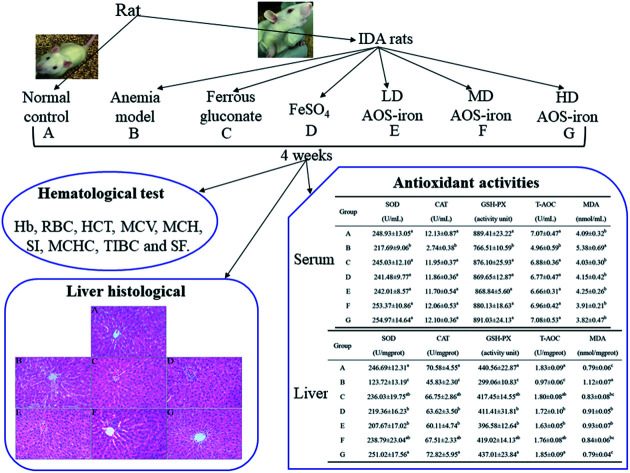

## 1 Introduction

Iron is an indispensable element for life, as it is an important component of human hemoglobin, cytochrome enzymes, and many reductases.^1^ Iron plays important roles in oxygen transportation, deoxyribonucleic acid (DNA) synthesis, mitochondrial electron transport, and ultimately in the entire energy metabolism.^2^ However, iron deficiency is very common in all age groups.^3^ Iron deficiency in the body can lead to iron deficiency anemia (IDA)^4^ and bodily dysfunction.^5^ Anemia occurs in one-third of the world's population and is mostly caused by iron deficiency.^6^ IDA is the commonest nutritional deficiency in the world and can affect mental and physical development,^7^ lead to an increase in lipid peroxidation and a decrease in antioxidant defenses,^8^ and trigger a decline in immune function^9^ and nervous system disorders.^10^

The most basic method for preventing IDA is to supply enough iron.^11^ Therefore, iron supplements with high bioavailability have been the focus of research. Ferrous sulfate has been permitted as an iron supplement in regulations related to pharmaceuticals and food additives in many countries, but it causes great gastrointestinal irritation.^12^ In recent years, ferrous gluconate and ferrous succinate have been used as organic iron supplements. However, the effectiveness of these supplements relies on the release of iron ions from gastric acid, and their absorptivity is relatively low in populations that lack stomach acid. Moreover, ferrous salts are unstable and difficult to produce and store.^13^ In order to increase iron utilization and reduce side effects, it is necessary to develop new iron supplements.

Studies of polysaccharide–iron complexes have been conducted. A polysaccharide–iron complex can effectively alleviate IDA *via* intravenous injection, but as an oral iron supplement it has less effect in comparison with ferrous sulfate.^14,15^ Therefore, oligosaccharide–iron complexes have aroused attention as oral iron supplements. Some published reports have indicated that oligosaccharide–iron complexes have exceptional water solubility and do not cause gastrointestinal irritation at oral doses.^16^ Mao *et al.*,^16^ Xu *et al.*^17^ and Yang *et al.*^18^ have studied the preparation, physicochemical properties and structural characteristics of isomaltooligosaccharide–iron, chitooligosaccharide–iron and soybean oligosaccharide–iron complexes, respectively. Isomaltooligosaccharide–iron complexes exhibited good reduction behavior and strong antioxidant activity *in vitro*.^16^ However, a study of the antioxidant activity of oligosaccharide–iron complexes *in vivo* has not been conducted. Agar oligosaccharide (AOS), which is a small-molecular sugar chain obtained by acid hydrolysis of agar from marine red algae, is an oligosaccharide that consists of alternating galactose and 3,6-*endo*-ether-galactose units. AOS has specific physiological functions, such as antioxidant,^19^ prebiotic,^20^ anti-inflammatory,^21^ and antimicrobial activity, glucosidase inhibition and melanin biosynthesis.^22^ In particular, AOS has high water solubility, is easily absorbed by the body, and contains a large number of free hydroxyl groups,^23,24^ which may coordinate with metal ions. Hence, AOS is a suitable choice as a ligand for chelating iron.

In this study, AOS was obtained by degrading agar polysaccharides, and an agar oligosaccharide–iron complex (AOS–iron) was prepared. A rat model of anemia was established to assess the effect of AOS–iron *in vivo*. The present study aimed to evaluate the effects of AOS–iron in terms of restoring iron status and promoting antioxidant activity by comparing the difference between the effects of ferrous gluconate, FeSO_4_, and AOS–iron on rats with IDA.

## 2 Materials and methods

### 2.1 Preparation of AOS–iron complex

AOS was obtained from agar (Fujian Lvqi Food Colloid Company, China) according to the method devised by Bartosz *et al.*^25^ and stored in a desiccant before use. AOS–iron was synthesized as described previously.^26^ The steps in the preparation of AOS–iron were as follows. First, 0.25 g AOS and 0.125 g sodium citrate were dissolved in 10 mL distilled water, and the pH was adjusted to 5.00 ± 0.05 with 1 mol L^−1^ HCl. Then, a 2.0 mol L^−1^ FeCl_3_ solution was added to the mixture in a mass ratio of AOS to iron ions of 4.5 : 1, followed by magnetic stirring at 74 °C for 60 min, and was then centrifuged for 15 min at 5500*g*. The supernatants were taken, concentrated under reduced pressure, and dialyzed with a dialysis bag (500 Da) for 48 h to eliminate unbound iron ions and other small molecules chelated by iron. The dialysate was freeze-dried and denoted as AOS–iron. The iron content of AOS–iron was 14.03%, as determined by atomic absorption spectrophotometry (AA-6300C, Shimadzu, Japan) with an air–acetylene flame.^27^

### 2.2 Animals and experimental design

#### 2.2.1 Animals

According to the requirement of the National Act on the Use of Experimental Animals (People's Republic of China), all experimental procedures involving animals closely followed the regulations of the Association for the Assessment and Accreditation of Laboratory Animal Care (AAALAC). All animal procedures were performed in accordance with the Guidelines for Care and Use of Laboratory Animals of Fujian Agriculture and Forestry University, and experiments were approved by the Animal Ethics Committee of the College of Food Science, Fujian Agriculture and Forestry University. Healthy male SPF Sprague-Dawley (SD) rats (*n* = 84) with an initial weight of 55 ± 5 g were obtained from Shanghai SLAC Laboratory Animal Co., Ltd (Shanghai, China). All rats were housed in stainless-steel cages (6 rats per cage) with sawdust bedding under controlled conditions of humidity (50 ± 10%), light (12 h/12 h light/dark cycle) and temperature (23 ± 2 °C). The sawdust bedding was renewed every three days, and the rats had free access to food and water.

#### 2.2.2 Experimental design

After being acclimatized for 5 days, the rats were randomly divided into a normal control group (A, *n* = 12) and an anemia model group (*n* = 72). The normal control group (A) was fed with a standard pelleted diet produced according to the American AIN93 standard (45 mg Fe per kg diet, Trophic Animal Feed High-tech Co., Ltd., Nantong, China) throughout the whole experimental period, whereas the anemia model group was fed with an iron-deficient diet produced according to the American AIN93 standard (12 mg Fe per kg diet, Trophic Animal Feed High-tech Co., Ltd, Nantong, China) to establish a model of iron deficiency anemia (IDA model). The whole experimental process was strictly controlled to avoid contamination with iron. From the second week, the blood hemoglobin (Hb) content was measured weekly. Blood samples were obtained from the orbits of the rats' eyes to determine the Hb content. In the fourth week, the Hb content was less than 70 g L^−1^, which indicated that the IDA model was established successfully.^28^

The IDA model rats were divided into six groups of 12 rats as follows: (B) an anemia model group (iron-deficient diet); (C) a ferrous gluconate group (iron-deficient diet + ferrous gluconate at a dose of 4 mg Fe per kg bw); (D) an FeSO_4_ group (iron-deficient diet + FeSO_4_ at a dose of 4 mg Fe per kg bw); and (E, F and G) low-dose (LD), medium-dose (MD) and high-dose (HD) AOS–iron groups (iron-deficient diet + AOS–iron at a dose of 1.0, 2.0, and 4.0 mg Fe per kg bw, respectively). Ferrous gluconate, FeSO_4_ and AOS–iron were dissolved in deionized water, and the rats received the abovementioned doses by intragastric administration. Rats in the anemia model group and normal control group were given the same volume of saline. All supplements were freshly prepared every day, and intragastric administration was performed each day at 9:00 AM for 4 weeks.

#### 2.2.3 Clinical observations

The features of all rats were observed daily, including the skin, fur, claws, tail, ears, nose, and eyes.

#### 2.2.4 Sample collection

During the entire experiment, food intake was recorded daily and body weights were measured weekly. At the end of the experimental period (8 weeks), the rats were fasted for 12 h, and blood samples were collected by cardiac puncture under anesthesia with 10% chloral hydrate. Part of the blood samples were collected into anticoagulant blood tubes (EDTA-K2) for immediate routine blood tests, and the other blood samples were transferred into blood collection tubes (without any additives) for the separation of serum. The serum was separated by cryogenic ultracentrifugation and stored at −80 °C for future analysis. Then all the rats were dissected, and the heart, liver, spleen, kidneys and testis were completely removed. The organs were rinsed with 0.9% physiological saline to remove residual blood from the surface of the organs and weighed. Part of the livers were stored in 10% neutral formalin for further histological analysis, and the other livers and all spleens were stored at −80 °C for further analysis of the iron content.

#### 2.2.5 Hematological tests

Hemoglobin (Hb), red blood cells (RBC), hematocrit (HCT), mean cell volume (MCV), mean cell hemoglobin (MCH) and mean cell hemoglobin concentration (MCHC) were measured with an automated hematology analyzer (Mindray BC-6800, Shenzhen, China). The serum iron (SI) concentration and total iron binding capacity (TIBC) were measured using an automatic biochemical analyzer (AU2700, Olympus, Tokyo, Japan). The serum ferritin (SF) concentration was measured with a microplate reader (Rayto RT-6100, Shanghai, China) and a rat ferritin enzyme-linked immunosorbent assay kit (Nanjing Jiancheng Bioengineering Inst., Nanjing, China). Transferrin saturation (TS) refers to the ratio of the SI concentration to the TIBC, which is calculated as follows:^29^1
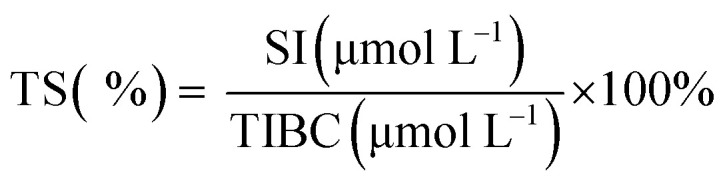


#### 2.2.6 Liver and spleen iron contents

Samples of 1.0 g liver and whole spleen were digested using a solution of an acid mixture (nitric acid : perchloric acid, 4 : 1, v/v) on a hot plate until clear solutions were obtained. Then the acid mixture was evaporated and diluted to 50.0 mL using deionized water. The iron contents in the liver and spleen were measured by atomic absorption spectrophotometry (AA-6300C, Shimadzu, Japan) with an air–acetylene flame.^27^

#### 2.2.7 Organ coefficients

The heart, liver, spleen, kidney and testis were weighed, and the weights were recorded. The organ coefficients were calculated by eqn (2):^30^2
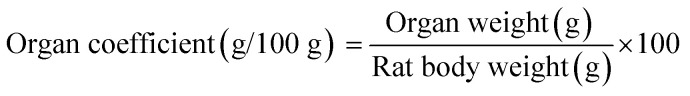


#### 2.2.8 Histological analysis

Liver tissues were fixed in 10% neutral formalin for 24–48 h, dehydrated by an ethanol gradient (70–100%), cleared by xylene, embedded in paraffin and cut into slices of 5 μm. Then the slices were stained with hematoxylin and eosin (HE), placed on a glass dish, sealed with a neutral gum, and examined under a light microscope (BA210T, Motic, China) at a magnification of 400×.^31^

#### 2.2.9 Determination of *in vivo* antioxidant activity

The activities of superoxide dismutase (SOD), catalase (CAT), and glutathione peroxidase (GSH-PX) and the total antioxidant capacity (T-AOC), as well as the malondialdehyde (MDA) content, in the serum and liver were determined using test kits (Nanjing Jiancheng Bioengineering Inst., Nanjing, China) according to the manufacturer's instructions.^32^

#### 2.2.10 Experimental design of AOS group for IDA model

Healthy male SPF SD rats (*n* = 36) with an initial weight of 55 ± 5 g were randomly divided into a normal control group (*n* = 12) and an IDA model group (*n* = 24). After the IDA model was established successfully, the IDA model rats were divided into an anemia model group (*n* = 12; iron-deficient diet) and an AOS group (*n* = 12; iron-deficient diet + AOS at a dose of 24.5 mg AOS per kg bw). The specific experimental steps and methods are described in Section 2.2.2. The Hb contents, RBC counts, SI concentration, and liver and spleen iron contents were determined as described in Sections 2.2.5 and 2.2.6.

#### 2.2.11 Statistical analysis

All quantitative results (numerical values and representative diagrams) were expressed as the mean ± standard deviation. Data were analyzed by ANOVA and Duncan's multiple-range tests using SPSS software (version 21.0; IBM Corp., Armonk, NY). A value of *p* < 0.05 was regarded as statistically significant.

## 3 Results and discussion

### 3.1 Body weight and growth status

**Fig. 1:**
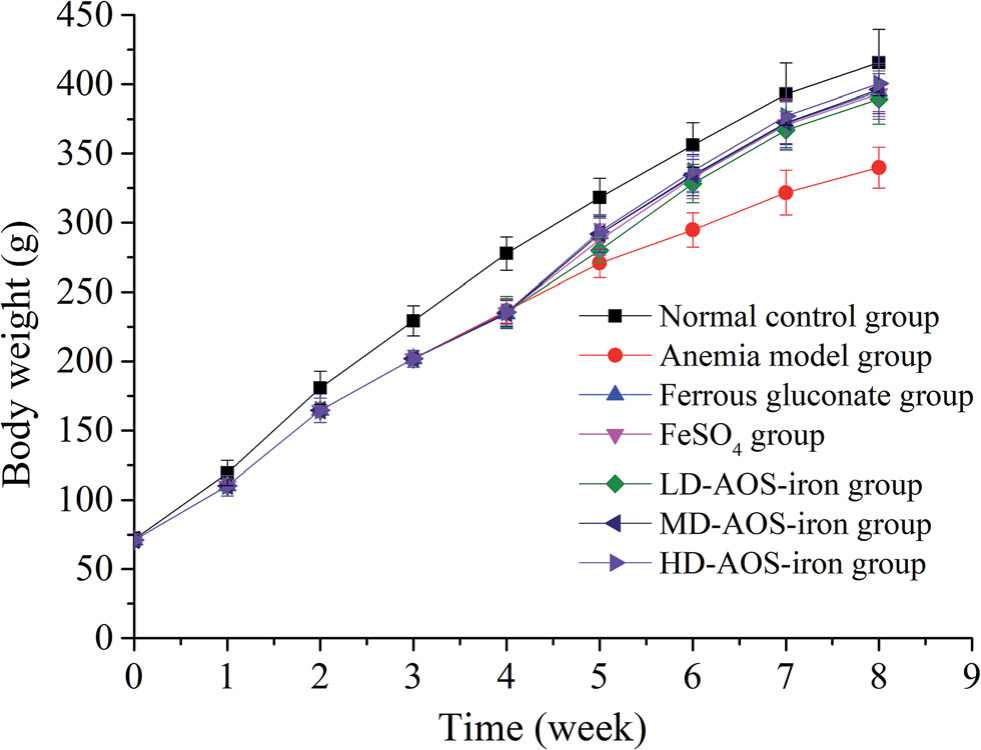
Changes in the body weights of the rats in the different groups: 0–4 weeks were the period of establishment of the IDA model, and 5–8 weeks were the period of iron supplementation.

IDA affects growth and development.^7^ As shown in Fig. 1, the body weights of the rats in the beginning (0 week) exhibited no significant differences (*p* > 0.05). In comparison with the normal control group, the body weights of the rats in the anemia model group displayed a significant decrease (*p* < 0.05) after feeding with the low-iron diet for 4 weeks, which was consistent with the study by Wang *et al.*^33^ After supplementation with iron, the body weights of the rats in all the iron supplementation groups significantly increased in comparison with the anemia model group (*p* < 0.05), whereas the differences in body weight between the ferrous gluconate, FeSO_4_, and AOS–iron groups were not significant (*p* > 0.05). At the end of iron supplementation (8 weeks), the body weights exhibited no significant differences (*p* > 0.05) between the HD AOS–iron group and the normal control group, but the other groups did not reach normal levels; this implied that AOS–iron more effectively increased the body weight of rats with IDA. During the modeling process, the skin, claws, ears, tails, noses and eyes of the rats gradually became pale, and the fur was rough and sparse. These phenomena became worse with an increase in the feeding time owing to the lack of iron intake.^34^ Whereas, the apparent performance of all the iron supplementation groups gradually improved and returned to normal levels. Some reports have confirmed that oligosaccharides can promote growth by enhancing the body's immune function.^35,36^ Hence, in comparison with ferrous gluconate and FeSO_4_, AOS–iron could greatly promote the growth of rats with IDA.

### 3.2 Routine blood tests

Blood mainly consists of tangible cells and body fluids. Routine blood tests are the most basic blood test methods used in clinics and are mainly used to test tangible cells in blood.^37^ Routine blood tests are often used to successfully diagnose IDA. The results of routine blood tests are shown in Table 1.

**Table 1:** Effect of iron supplementation on results of routine blood tests on rats in different groups^a^

Group	Dosage (mg Fe per kg bw)	Hb (g L^−1^)	RBC (10^12^/L)	HCT (%)	MCV (fL)	MCH (pg)	MCHC (g L^−1^)
Normal control	0	158.08 ± 5.87^a^	8.46 ± 0.40^a^	49.92 ± 2.43^a^	60.34 ± 1.61^a^	19.73 ± 0.69^a^	320.33 ± 3.75^a^
Anemia model	0	41.11 ± 2.90^c^	2.21 ± 0.66^c^	10.67 ± 2.87^d^	46.98 ± 2.14^d^	16.67 ± 1.10^d^	251.44 ± 2.46^c^
Ferrous gluconate	4	154.58 ± 6.75^ab^	8.38 ± 0.37^ab^	47.83 ± 3.07^ab^	57.12 ± 1.72^b^	19.06 ± 0.90^ab^	319.83 ± 4.26^ab^
FeSO_4_	4	152.91 ± 4.25^b^	8.06 ± 0.29^b^	45.82 ± 3.84^b^	56.84 ± 2.51^b^	18.83 ± 0.76^b^	316.18 ± 3.37^b^
LD-AOS–iron	1	149.25 ± 5.80^b^	7.98 ± 0.41^b^	42.92 ± 3.29^c^	51.32 ± 3.38^c^	17.93 ± 1.19^c^	314.17 ± 6.21^b^
MD-AOS–iron	2	157.33 ± 6.02^ab^	8.42 ± 0.36^ab^	49.08 ± 1.68^a^	57.30 ± 1.37^b^	19.36 ± 1.26^ab^	320.25 ± 3.41^a^
HD-AOS–iron	4	158.58 ± 4.54^a^	8.48 ± 0.52^a^	49.83 ± 3.01^a^	59.73 ± 2.20^a^	19.74 ± 0.61^a^	323.58 ± 6.05^a^

a The results are presented as the mean ± SD (*n* = 12). Different lower-case roman letters in the same column indicate a significant difference between different groups (*p* < 0.05).

Hemoglobin (Hb) is the main constituent of red blood cells, which carry oxygen to various body parts for utilization by tissue.^38^ As shown in Table 1, at the end of the experiment (8 weeks), the Hb content (41.11 ± 2.90 g L^−1^) in the anemia model group was significantly lower (*p* < 0.05) than that in the normal control group. The reason was that iron deficiency led to a decrease in functional iron in circulation in the blood, which resulted in a decrease in the Hb content.^39,40^ Moreover, the Hb contents in the HD AOS–iron group and ferrous gluconate group were similar (*p* > 0.05) and attained normal levels, whereas the Hb content in the FeSO_4_ group was significantly lower than that in the normal control group (*p* < 0.05). Iron is indispensable for the synthesis of Hb in red blood cells.^41^ Therefore, our results confirmed that iron supplements could increase the blood Hb content; in particular, HD AOS–iron had a good recovery effect on the Hb content. In comparison with the recovery effect of a polysaccharide–iron complex on the Hb content, the same dose of AOS–iron led to a better improvement.^42^ This result suggested that an oligosaccharide ligand in an iron complex can increase the bioavailability of iron to a much greater extent than a polysaccharide ligand.

Red blood cells (RBC), which contain 90% Hb, are the most abundant cells suspended in blood.^43^ They play a crucial role in the transportation of gases in the body, and a sufficient number of RBC is necessary to maintain the normal acid–base balance of the body.^44^ The hematocrit (HCT) is defined as the ratio of RBC volume to whole blood volume.^38^Table 1 shows that RBC count and HCT value in the anemia model group were significantly lower than those in the normal control group (*p* < 0.05). Because RBC and HCT are positively correlated with Hb,^32,38^ iron deficiency led to a decrease in the Hb content (see Table 1), which caused the decline in the numbers of RBC and HCT values in the rats with IDA. In contrast, the RBC counts and HCT values in all the iron supplementation groups were significantly higher (*p* < 0.05) in comparison with the anemia model group, and the RBC counts and HCT values in the ferrous gluconate group and MD and HD AOS–iron groups increased to normal levels (*p* < 0.05), in contrast to the FeSO_4_ group and LD AOS–iron group. Our results indicated that MD AOS–iron was sufficient to increase the RBC count and HCT value in rats with anemia to normal levels.

The mean cell volume (MCV) refers to the average volume of RBC, which reflects the average size of RBC. The mean cell hemoglobin (MCH) and mean cell hemoglobin concentration (MCHC) reflect the Hb content of RBC.^45^ Hence, MCV, MCH and MCHC are collectively referred to as the average red blood cell index.^46^ As shown in Table 1, MCV, MCH and MCHC in the anemia model group were significantly reduced (*p* < 0.05) in comparison with the normal control group. In fact, owing to iron deficiency, the Hb content in RBC was reduced, which resulted in a decrease in cell volumes.^47^ After iron supplementation, these parameters in the iron supplementation groups significantly increased in comparison with those in the anemia model group (*p* < 0.05). The values of MCV in the MD AOS–iron group, ferrous gluconate group and FeSO_4_ group were significantly lower than that in the normal group (*p* < 0.05), and only those in the HD AOS–iron group recovered to normal levels (*p* < 0.05). Except for those in the FeSO_4_ group and LD AOS–iron group, the values of MCH and MCHC exhibited no significant differences (*p* > 0.05) between the iron supplementation groups and the normal control group. On the basis of the above results and analysis, HD AOS–iron can fully restore the cell size (MCV) and Hb content (MCH and MCHC) of RBC in rats with IDA.

### 3.3 SI, TIBC and TS levels

The serum iron (SI) concentration is the total amount of iron in serum. The total iron binding capacity (TIBC) refers to the maximum amount of iron required to bind to all the transferrin in serum. The TIBC increases when the SI concentration is low and decreases when the SI concentration is high.^48^ Transferrin saturation (TS) refers to the amount of iron in bound form.

The changes in SI, TIBC, and TS levels are shown in Table 2. The anemia model group exhibited significantly lower SI and TS levels and higher TIBC levels in comparison with the normal control group (*p* < 0.05) because it was always fed with a low-iron diet.^34^ It is noteworthy that the SI and TS levels in the ferrous gluconate, FeSO_4_ and AOS–iron groups were significantly higher than those in the anemia model group (*p* < 0.05) and were similar (*p* > 0.05). In contrast, the TIBC levels in the ferrous gluconate, FeSO_4_, and MD and HD AOS–iron groups were not significantly different in comparison with the normal control group (*p* > 0.05) but were significantly lower than those in the anemia model group (*p* < 0.05). Usually, SI, TS and TIBC reflect the status of iron during its circulation in the blood.^49^ Attractive results included the fact that MD AOS–iron restored the SI, TIBC, and TS levels to normal levels and exhibited a better effect than traditional iron supplements. Therefore, AOS–iron was more effective in improving the blood circulation index, *i.e.*, it was more beneficial for increasing the body's level of “transport iron”.^50^

**Table 2:** Effect of iron supplementation on SI, TIBC and TS of rats in different groups^a^

Group	Dosage (mg Fe per kg bw)	SI (μmol L^−1^)	TIBC (μmol L^−1^)	TS (%)
Normal control	0	27.15 ± 2.05^a^	72.55 ± 4.84^c^	37.43 ± 2.45^ab^
Anemia model	0	3.83 ± 0.30^b^	113.92 ± 5.32^a^	3.37 ± 0.32^c^
Ferrous gluconate	4	28.72 ± 3.48^a^	71.83 ± 4.24^c^	40.04 ± 4.78^a^
FeSO_4_	4	27.55 ± 2.94^a^	72.91 ± 3.27^c^	37.90 ± 4.88^ab^
LD-AOS–iron	1	27.08 ± 4.10^a^	76.83 ± 6.35^b^	35.45 ± 6.38^b^
MD-AOS–iron	2	28.16 ± 2.33^a^	71.92 ± 2.84^c^	39.19 ± 3.44^ab^
HD-AOS–iron	4	29.14 ± 3.93^a^	70.67 ± 4.46^c^	41.30 ± 5.51^a^

a The results are presented as the mean ± SD (*n* = 12). Different lower-case roman letters in the same column indicate a significant difference between different groups (*p* < 0.05).

### 3.4 SF content

Serum ferritin (SF) is a complex formed by apoferritin and an iron core (Fe^3+^) that has a high capacity to bind and store iron to maintain the relative stability of the iron supply and Hb content in the body.^51^ Hence, the SF content is always considered to be a sensitive index of the status of body iron stores.^52^

**Fig. 2:**
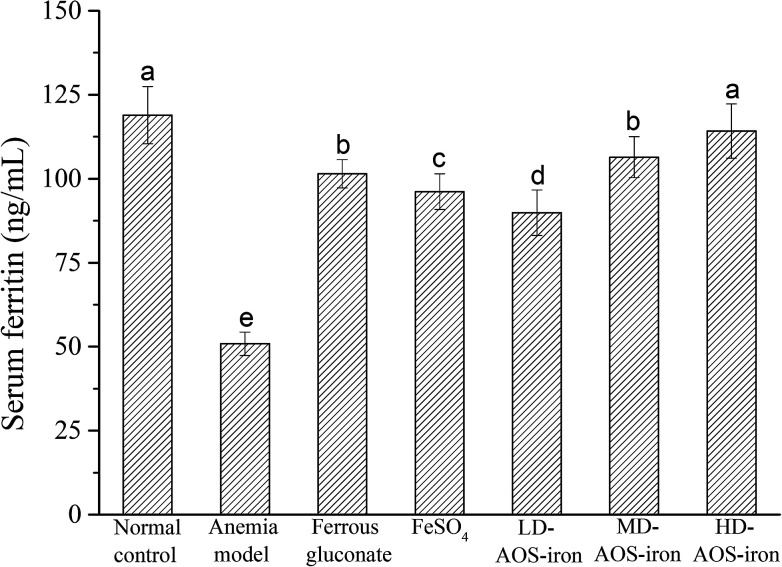
Effect of iron supplementation on the SF content in rats in different groups. Different lower-case roman letters indicate statistically significant differences (*p* < 0.05).

As shown in Fig. 2, the SF content in the anemia model group was lower than that in the normal control group (*p* < 0.05). The SF content was lowest in the anemia model group (50.88 ± 3.47 ng mL^−1^) and highest in the normal group (118.94 ± 8.51 ng mL^−1^), which was consistent with the report by Wang *et al.*^33^ The SF contents in all the iron supplementation groups were significantly higher (*p* < 0.05) in comparison with that in the anemia model group. Although all the iron supplements significantly restored the SF content in the rats with IDA (*p* < 0.05), only HD AOS–iron increased the SF content to normal levels. Normally, a positive relationship exists between the SF content and the amount of iron stored in the body.^53^ Hence, our results further indicated that HD AOS–iron more effectively increased the amount of iron stored in the body than traditional iron supplements.

### 3.5 Liver and spleen iron contents

**Fig. 3:**
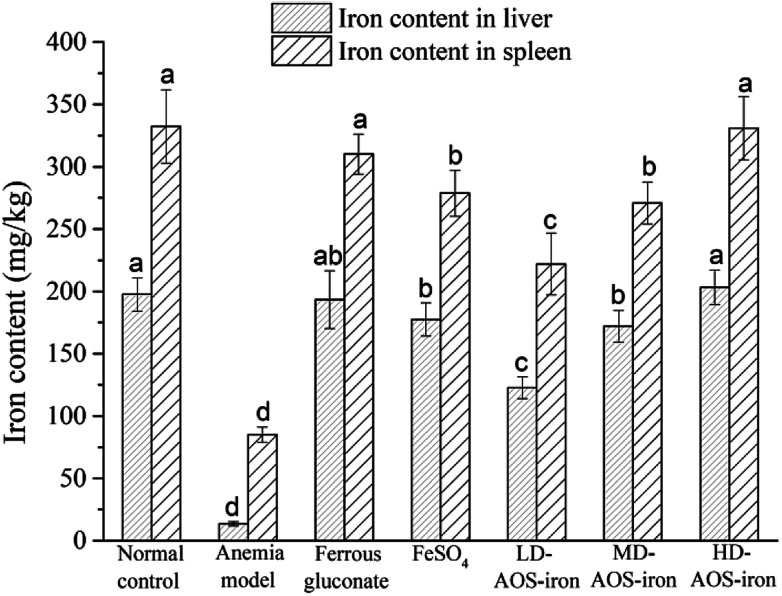
Iron contents of liver and spleen of rats in different groups. Different lower-case roman letters indicate statistically significant differences (*p* < 0.05).

The liver and spleen are the main organs of iron storage and play an important role in iron metabolism.^54^Fig. 3 shows that the iron contents of the liver and spleen in the anemia model group were significantly lower (*p* < 0.05) than those in the normal control group. In comparison with the anemia model group, the iron contents of the liver and spleen were significantly increased by supplementation with ferrous gluconate, FeSO_4_ and AOS–iron. Even though the iron contents of the liver and spleen were increased by different degrees by the various iron supplements, only the iron contents of the liver and spleen in the HD AOS–iron and ferrous gluconate groups recovered to normal levels.

In addition to functional iron and transport iron in the body, the entire body contains 25–30% storage iron.^55^ Ferritin is an iron storage protein that is mainly found in the liver and spleen in mammals.^56^ Ferritin can release iron at times of iron deficiency in the blood circulation. In order to maintain the SI concentration and the synthesis of Hb, iron stores in the liver and spleen are mobilized to respond to the body's demands.^57^ Therefore, the low iron contents of the liver and spleen in rats with IDA indicated severe depletion of storage iron, which was similar to the results of a study by Kasai *et al.*^58^ With the same dose of the iron supplement, the recovery effects of HD AOS–iron on the iron contents of the liver and spleen were comparable to those of ferrous gluconate and superior to those of inorganic iron (FeSO_4_). A possible reason was that AOS promoted the absorption of iron by regulating the intestinal flora.^59^

### 3.6 Organ coefficients

The measurement of organ coefficients is an important part of the evaluation of drug safety that is used to reflect the degree of internal organ disease.^60^Table 3 shows the organ coefficients for rats in different groups. The heart coefficient in the anemia model group was significantly higher than that in the normal control group (*p* < 0.05), which indicated that iron deficiency led to cardiac hypertrophy.^61^ The heart coefficients in all the iron supplementation groups were significantly reduced (*p* < 0.05), and only HD AOS–iron restored the heart coefficient to normal levels. A significant decrease in the liver coefficient was observed in the anemia model group, which was consistent with the result reported by Zhang *et al.*^34^ and indicated that iron deficiency caused a decrease in liver volume. A possible reason is that, when there is iron deficiency, DNA synthesis in the liver is inhibited, which results in slower development of the liver.^62^ After iron supplementation, the liver coefficients in all the iron supplementation groups significantly increased (*p* < 0.05) and exhibited no significant differences in comparison with that in the normal control group (*p* > 0.05). Spleen hypertrophy was observed in the anemia model group, for which the reason was that iron deficiency resulted in cell proliferation by activating spleen cells.^63^ The three kinds of iron supplement reversed the spleen hypertrophy to a normal status. Furthermore, the organ coefficients of the kidney and testis in the anemia model group were significantly higher in comparison with those in the normal control group (*p* < 0.05). The swelling of the kidney and testis might have resulted from edema caused by iron deficiency.^64^ However, the coefficients of the kidney and testis in all the iron supplementation groups exhibited no significant differences in comparison with those in the normal control group (*p* > 0.05). In general, a high dosage of AOS–iron could restore the statuses of all organs and had a particular advantage for the heart.

**Table 3:** Organ coefficients of the heart, liver, spleen, kidney and testis of rats in different groups^a^

Group	Dosage (mg Fe per kg bw)	Organ coefficient (g/100 g)				
		Heart	Liver	Spleen	Kidney	Testis
Normal control	0	0.31 ± 0.04^c^	2.72 ± 0.25^a^	0.21 ± 0.05^b^	0.67 ± 0.05^b^	0.90 ± 0.07^b^
Anemia model	0	0.57 ± 0.06^a^	2.41 ± 0.16^b^	0.43 ± 0.09^a^	0.75 ± 0.06^a^	1.07 ± 0.09^a^
Ferrous gluconate	4	0.35 ± 0.04^bc^	2.64 ± 0.25^a^	0.23 ± 0.02^b^	0.67 ± 0.08^b^	0.95 ± 0.08^b^
FeSO_4_	4	0.37 ± 0.03^b^	2.63 ± 0.15^a^	0.22 ± 0.03^b^	0.69 ± 0.09^ab^	0.94 ± 0.06^b^
LD-AOS–iron	1	0.38 ± 0.04^b^	2.59 ± 0.16^a^	0.23 ± 0.03^b^	0.70 ± 0.08^ab^	0.95 ± 0.06^b^
MD-AOS–iron	2	0.36 ± 0.02^b^	2.64 ± 0.21^a^	0.23 ± 0.04^b^	0.68 ± 0.08^ab^	0.93 ± 0.05^b^
HD-AOS–iron	4	0.32 ± 0.02^c^	2.67 ± 0.24^a^	0.22 ± 0.03^b^	0.65 ± 0.07^b^	0.89 ± 0.05^b^

a The results are presented as the mean ± SD (*n* = 12). Different lower-case roman letters in the same column indicate a significant difference between different groups (*p* < 0.05).

### 3.7 Histological analysis of the liver

**Fig. 4:**
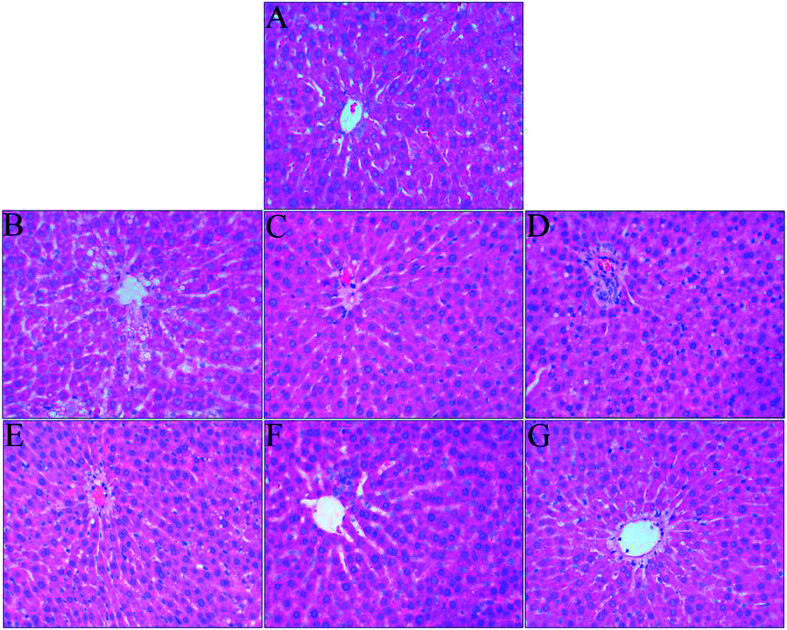
Effect of iron supplementation on histomorphological changes in the liver in rats (HE staining, 400× magnification): (A) normal control group; (B) anemia model group; (C) ferrous gluconate group; (D) FeSO_4_ group; (E) LD-AOS–iron group; (F) MD-AOS–iron group; (G) HD-AOS–iron group.

The liver is an important organ of iron metabolism in the body.^65^ The structures of liver tissues stained with HE are shown in Fig. 4. The liver cells in normal rats were arranged in single rows radially from the central vein, the morphology of hepatocytes was complete, and the nuclei were clear (Fig. 4A). A photomicrograph of liver cells from the anemia model group (Fig. 4B) shows that the boundaries of liver plates were not clear, the arrangement of hepatic sinusoids was disordered and slightly expanded, and massive fat vacuoles of various sizes can also be seen in the hepatocytes. In the ferrous gluconate group (Fig. 4C), the hepatocytes were complete, the nuclei were large and roundish, and there were hardly any fat vacuoles in the hepatocytes, but the liver plates were slightly dilated. The hepatocytes of the rats in the FeSO_4_ group were complete (Fig. 4D), but the boundaries of liver plates were unclear, the hepatic sinusoids were disordered, and there was a small amount of fat vacuoles in the hepatocytes. With an increase in the iron dose in AOS–iron, the degree of damage to the livers of rats with IDA was gradually reversed to a normal status (Fig. 4E–G). Photomicrographs of liver cells from the MD and HD AOS–iron groups indicated that the liver plates around the central vein were clear and arranged in an orderly manner and there were hardly any fat vacuoles in the hepatocytes.

The disarrangement of the hepatic plates and hepatic sinusoids might have been due to the inhibition of DNA synthesis in the liver caused by IDA.^62^ Furthermore, when there was iron deficiency, lipid metabolism in the liver was disordered,^66^ which led to the appearance of fat vacuoles in the hepatocytes. After iron supplementation, the disordered arrangement of hepatic sinusoids and liver plates and the presence of fat vacuoles in the hepatocytes were reversed to different degrees, and photomicrographs of liver cells from the MD and HD AOS–iron groups were similar to those for the normal group. Importantly, the effect of AOS–iron in repairing liver tissue was better than those of ferrous gluconate and FeSO_4_.

### 3.8 Antioxidant activities in serum and liver

The body's antioxidant enzyme system is mainly composed of SOD, CAT and GSH-PX. SOD is the first line of defense against the body's reactive oxygen species (ROS), CAT is a key enzyme in the biological defense system, and GSH-PX plays a protective role against oxidation of lipid membranes.^67^ SOD catalyzes the disproportionation of superoxide radicals to hydrogen peroxide (H_2_O_2_), whereas CAT and GSH-PX convert H_2_O_2_ to H_2_O and thereby protect cells against damage due to ROS.^68^ The T-AOC reflects the total antioxidant capacity of the body's defense system,^68^ and the MDA content reflects the degree of lipid peroxidation in the body.^69^

The results for the antioxidant activities in the serum and liver are shown in Tables 4 and 5, respectively. In comparison with the normal control group, the activities of SOD, CAT, and GSH-PX and the T-AOC were significantly lower (*p* < 0.05) and the MDA content was significantly higher (*p* < 0.05) in the serum and liver in the anemia model group. Therefore, IDA resulted in the accumulation of ROS and caused oxidative stress (OS) by disrupting the balance of the antioxidant enzyme system.^70,71^

**Table 4:** Effect of iron supplementation on antioxidant activity in serum of rats in different groups^a^

Group	Dosage (mg Fe per kg bw)	SOD (U mL^−1^)	CAT (U mL^−1^)	GSH-PX (activity units)	T-AOC (U mL^−1^)	MDA (nmol mL^−1^)
Normal control	0	248.93 ± 13.05^a^	12.13 ± 0.87^a^	889.41 ± 23.22^a^	7.07 ± 0.47^a^	4.09 ± 0.32^b^
Anemia model	0	217.69 ± 9.06^b^	2.74 ± 0.38^b^	766.51 ± 10.59^b^	4.96 ± 0.59^b^	5.38 ± 0.69^a^
Ferrous gluconate	4	245.03 ± 12.10^a^	11.95 ± 0.37^a^	876.10 ± 25.93^a^	6.88 ± 0.36^a^	4.03 ± 0.30^b^
FeSO_4_	4	241.48 ± 9.77^a^	11.86 ± 0.36^a^	869.65 ± 12.87^a^	6.77 ± 0.47^a^	4.15 ± 0.42^b^
LD-AOS–iron	1	242.01 ± 8.57^a^	11.70 ± 0.54^a^	868.84 ± 5.60^a^	6.66 ± 0.31^a^	4.25 ± 0.26^b^
MD-AOS–iron	2	253.37 ± 10.86^a^	12.06 ± 0.53^a^	880.13 ± 18.63^a^	6.96 ± 0.42^a^	3.91 ± 0.21^b^
HD-AOS–iron	4	254.97 ± 14.64^a^	12.10 ± 0.36^a^	891.03 ± 24.13^a^	7.08 ± 0.53^a^	3.82 ± 0.47^b^

a The results are presented as the mean ± SD (*n* = 12). Different lower-case roman letters in the same column indicate a significant difference between different groups (*p* < 0.05).

**Table 5:** Effect of iron supplementation on antioxidant activity in liver of rats in different groups^a^

Group	Dosage (mg Fe per kg bw)	SOD (U per mg prot)	CAT (U per mg prot)	GSH-PX (activity units)	T-AOC (U per mg prot)	MDA (nmol mg^−1^ prot)
Normal control	0	246.69 ± 12.31^a^	70.58 ± 4.55^a^	440.56 ± 22.87^a^	1.83 ± 0.09^a^	0.79 ± 0.06^c^
Anemia model	0	123.72 ± 13.19^c^	45.83 ± 2.30^c^	299.06 ± 10.83^c^	0.97 ± 0.06^c^	1.12 ± 0.07^a^
Ferrous gluconate	4	236.03 ± 19.75^ab^	66.75 ± 2.86^ab^	417.45 ± 14.55^ab^	1.80 ± 0.08^ab^	0.83 ± 0.08^bc^
FeSO_4_	4	219.36 ± 16.23^b^	63.62 ± 3.50^b^	411.41 ± 31.81^b^	1.72 ± 0.10^b^	0.91 ± 0.05^b^
LD-AOS–iron	1	207.67 ± 17.02^b^	60.11 ± 4.74^b^	396.58 ± 12.64^b^	1.63 ± 0.05^b^	0.93 ± 0.07^b^
MD-AOS–iron	2	238.79 ± 23.04^ab^	67.51 ± 2.33^ab^	419.02 ± 14.13^ab^	1.76 ± 0.08^ab^	0.84 ± 0.06^bc^
HD-AOS–iron	4	251.02 ± 17.56^a^	72.82 ± 5.95^a^	437.01 ± 23.84^a^	1.85 ± 0.09^a^	0.79 ± 0.04^c^

a The results are presented as the mean ± SD (*n* = 12). Different lower-case roman letters in the same column indicate a significant difference between different groups (*p* < 0.05).

At the end of iron supplementation, the activities of SOD, CAT, and GSH-PX and the T-AOC significantly increased (*p* < 0.05) and the MDA content was significantly reduced (*p* < 0.05) in all the iron supplementation groups in comparison with the anemia model group. As shown in Table 4, there were no significant differences in the activities of SOD, CAT, and GSH-PX, the T-AOC and the MDA content in serum between the iron supplementation groups (*p* > 0.05), and all of these parameters reached normal levels. As shown in Table 5, there were no significant differences in the activities of SOD, CAT, and GSH-PX, the T-AOC and the MDA content in the liver between the ferrous gluconate group and the FeSO_4_ group (*p* > 0.05). The activities of SOD, CAT, and GSH-PX and the T-AOC in the livers from the AOS–iron group increased with an increase in the dosage, whereas the MDA content displayed the opposite trend. In particular, MD and HD AOS–iron could restore the antioxidant activity in the livers to a normal status.

As can be seen, the change trends in the antioxidant activity in serum in all groups were consistent with those in the SI level (see Table 2), and the change trends in the antioxidant activity in the liver in all groups were consistent with those in the iron content of the liver (see Fig. 3). The results also confirmed that the antioxidant activities were positively correlated with the iron contents of the serum and liver. These results indicated that iron can improve antioxidant activity in an organism.

Our results indicated that IDA could decrease the activities of SOD, CAT, and GSH-PX and the T-AOC and increase the MDA content. MD and HD AOS–iron significantly increased the activity of antioxidant enzymes and decreased the MDA content to normal levels, which indicated that AOS–iron was more effective than traditional iron supplements and suggested that AOS–iron could alleviate the damage to cells due to IDA and lipid peroxidation and reduce the degree of damage due to OS in the body.^71^ In addition, the published literature indicates that AOS could significantly improve the activity of antioxidant enzymes and reduce the MDA content and thus exhibit high antioxidant capacity.^72^ Specifically, AOS could effectively protect cells from oxidatively induced death by eliminating ROS-induced intracellular oxidative damage.^72^ In short, AOS may also play an important part in restoring the function of the antioxidant system in rats with IDA.

### 3.9 Effect of AOS on IDA

In order to investigate if AOS had a beneficial effect on iron metabolism in IDA, animal experiments were conducted. The results for typical indices are shown in Table 6. In comparison with the normal control group, the anemia model group and AOS group exhibited significant decreases in the contents of Hb, RBC, SI, and liver and spleen iron (*p* < 0.05). In particular, there were no significant differences in the contents of Hb, RBC, SI, and liver and spleen iron between the anemia model group and the AOS group (*p* > 0.05), which indicated that supplying AOS alone to rats with IDA did not increase the contents of Hb, RBC, SI, and liver and spleen iron. In addition, in comparison with the HD AOS–iron group (Tables 1 and 2 and Fig. 3), the contents of Hb, RBC, SI, and liver and spleen iron were significantly lower in the AOS group even at the same supplemental dose of AOS. Therefore, our results suggested that AOS acted only as a ligand in AOS–iron, and AOS–iron was more conducive to absorption.

**Table 6:** Effects of AOS on contents of Hb, RBC, SI, and liver and spleen iron in rats with IDA^a^

Group	Dosage (mg AOS per kg bw)	Hb (g L^−1^)	RBC (10^12^/L)	SI (μmol L^−1^)	Iron content in liver (mg kg^−1^)	Iron content in spleen (mg kg^−1^)
Normal control	0	157.83 ± 7.12^a^	8.52 ± 0.43^a^	26.75 ± 3.20^a^	193.40 ± 15.89^a^	338.21 ± 25.25^a^
Anemia model	0	42.83 ± 5.46^b^	2.25 ± 0.50^b^	3.78 ± 0.49^b^	16.48 ± 1.67^b^	82.44 ± 8.59^b^
AOS	24.5	44.25 ± 5.67^b^	2.22 ± 0.48^b^	4.09 ± 0.55^b^	15.82 ± 1.96^b^	86.11 ± 6.36^b^

a The results are presented as the mean ± SD (*n* = 12). Different lower-case roman letters in the same column indicate a significant difference between different groups (*p* < 0.05).

## 4 Conclusions

The present study demonstrated that a novel complex, namely, AOS–iron, can significantly promote body growth, increase blood indices, organ iron contents and the main organ coefficients in rats with IDA to normal levels, and restore liver tissue to a normal status. Moreover, AOS–iron could enhance the activities of antioxidant enzymes and reduce the MDA content in rats with IDA to normal levels. AOS–iron exhibited better effects in terms of iron supplementation and the recovery of antioxidant ability than the traditional iron supplements like ferrous gluconate and FeSO_4_. AOS–iron might be used as an effective novel iron supplement to treat IDA. The mechanism whereby AOS–iron alleviates IDA and promotes antioxidant activities will be further studied.

## Conflicts of interest

The authors declare no competing financial interests.

## Supplementary Material
